# Seasonality of the bacterial and archaeal community composition of the Northern Barents Sea

**DOI:** 10.3389/fmicb.2023.1213718

**Published:** 2023-07-07

**Authors:** Stefan Thiele, Anna Vader, Stuart Thomson, Karoline Saubrekka, Elzbieta Petelenz, Oliver Müller, Gunnar Bratbak, Lise Øvreås

**Affiliations:** ^1^Department of Biological Science, University of Bergen, Bergen, Norway; ^2^Bjerknes Centre for Climate Research, Bergen, Norway; ^3^University Center in Svalbard (UNIS), Longyearbyen, Norway; ^4^Department of Bioscience, University of Oslo, Oslo, Norway

**Keywords:** microbial ecology, Arctic, the Nansen Legacy, polar microbes, carbon cycling, nitrogen cycling

## Abstract

The Barents Sea is a transition zone between the Atlantic and the Arctic Ocean. The ecosystem in this region is highly variable, and a seasonal baseline of biological factors is needed to monitor the effects of global warming. In this study, we report the results from the investigations of the bacterial and archaeal community in late winter, spring, summer, and early winter along a transect through the northern Barents Sea into the Arctic Ocean east of Svalbard using 16S rRNA metabarcoding. Winter samples were dominated by members of the SAR11 clade and a community of nitrifiers, namely *Cand*. Nitrosopumilus and LS-NOB (*Nitrospinia*), suggest a prevalence of chemoautotrophic metabolisms. During spring and summer, members of the *Gammaproteobacteria* (mainly members of the SAR92 and OM60(NOR5) clades, *Nitrincolaceae*) and *Bacteroidia* (mainly *Polaribacter, Formosa*, and members of the NS9 marine group), which followed a succession based on their utilization of different phytoplankton-derived carbon sources, prevailed. Our results indicate that Arctic marine bacterial and archaeal communities switch from carbon cycling in spring and summer to nitrogen cycling in winter and provide a seasonal baseline to study the changes in these processes in response to the effects of climate change.

## 1. Introduction

As an effect of global warming, the Arctic warms four times faster than the global average (Rantanen et al., 2022). In the Arctic, the Barents Sea is a transition zone between the Atlantic and the Arctic Ocean. In this study, warm Atlantic water mixes with cold Artic water over a shallow shelf area. In the northern Barents Sea, the effects of the influx of Atlantic water masses are, among others, increased productivity and the potential influx of phytoplankton blooms (Ingvaldsen et al., 2021), which may have profound effects on the highly dynamic marine ecosystems of the Arctic (Loeng, 1991; Reigstad et al., 2002; Wassmann et al., 2011; Smedsrud et al., 2013). Seasonal changes between the dark winter and the light summer are extreme, and the harsh environmental conditions during winter have made most sampling campaigns in the Arctic focused on the light season. Due to its high variability, seasonal baselines for the biological components of the ecosystem are necessary to investigate changes and predict the consequences of global warming. The aim of the Nansen Legacy project is to provide such a baseline in a holistic approach, including the microbial loop and the bacterial and archaeal communities.

During spring, phytoplankton blooms occur, and the primary production increases in the open water and under the sea ice in the Barents Sea and the Arctic Ocean (Sakshaug, 2004; Hodal and Kristiansen, 2008; Ardyna et al., 2020). Such spring blooms are the main source of carbon in marine ecosystems and vary in strength depending on freshwater influx due to sea ice melt in the Northern Barents Sea (Hunt et al., 2013; Silva et al., 2021). These blooms are followed by successions of mostly heterotrophic bacteria such as *Bacteroidia, Gammaproteobacteria*, and *Alphaproteobacteria* (*Rhodobacteraceae)*, based on the predominant carbon sources (Teeling et al., 2012, 2016; de Sousa et al., 2019; Wietz et al., 2021; Thiele et al., 2023). Therefore, during summer, the communities are dominated by *Gammaproteobacteria* (*Nitrincolaceae*, members of the SAR92 and OM60(NOR5) clades, *Halomonadaceae, Pseudohongiellacea*, and *Alteromonadaceae*), *Bacteroidetes* (*Polaribacter, Formosa*, and *Ulvibacter*), and *Alphaproteobacteria* (members of the SAR11 clades, *Rhodobacteraceae*, and *Roseobacter*). The exploitation of phytoplankton-derived carbon remains dominant until autumn with different bacterial groups, e.g., *Flavobacteriaceae* and *Rhodobacteraceae*, dominating the carbon decomposition at different times. Other groups, such as *Nitrosphaerota* (*Candidatus* Nitrosopumilus), *Marinimicrobia* (SAR406 clade), and *Chloroflexi* (members of the SAR202 clade), are abundant in sub-seasurface waters during all seasons (Alonso-Sáez et al., 2012; Grzymski et al., 2012; Wilson et al., 2017; Müller et al., 2018; Wietz et al., 2021; Thiele et al., 2023). During the winter months, ammonia oxidation by *Nitrosphaerota* (formerly associated with *Thaumarchaea*), namely *Cand*. Nitrosopumilus becomes a major metabolic process in the surface and sub-seasurface waters. This results in the replenishment of the nitrate concentrations in surface waters, where it is taken up by the phytoplankton in spring (Grzymski et al., 2012; Connelly et al., 2014).

In this study, we present an investigation of the bacterial and archaeal community of a transect from the northern Barents Sea into the Arctic Ocean during late winter, spring, summer, and early winter setting a baseline for future investigations with regard to global warming-induced changes and their effect on the microbial community.

## 2. Materials and methods

### 2.1. Sampling and environmental parameters

**Figure 1:**
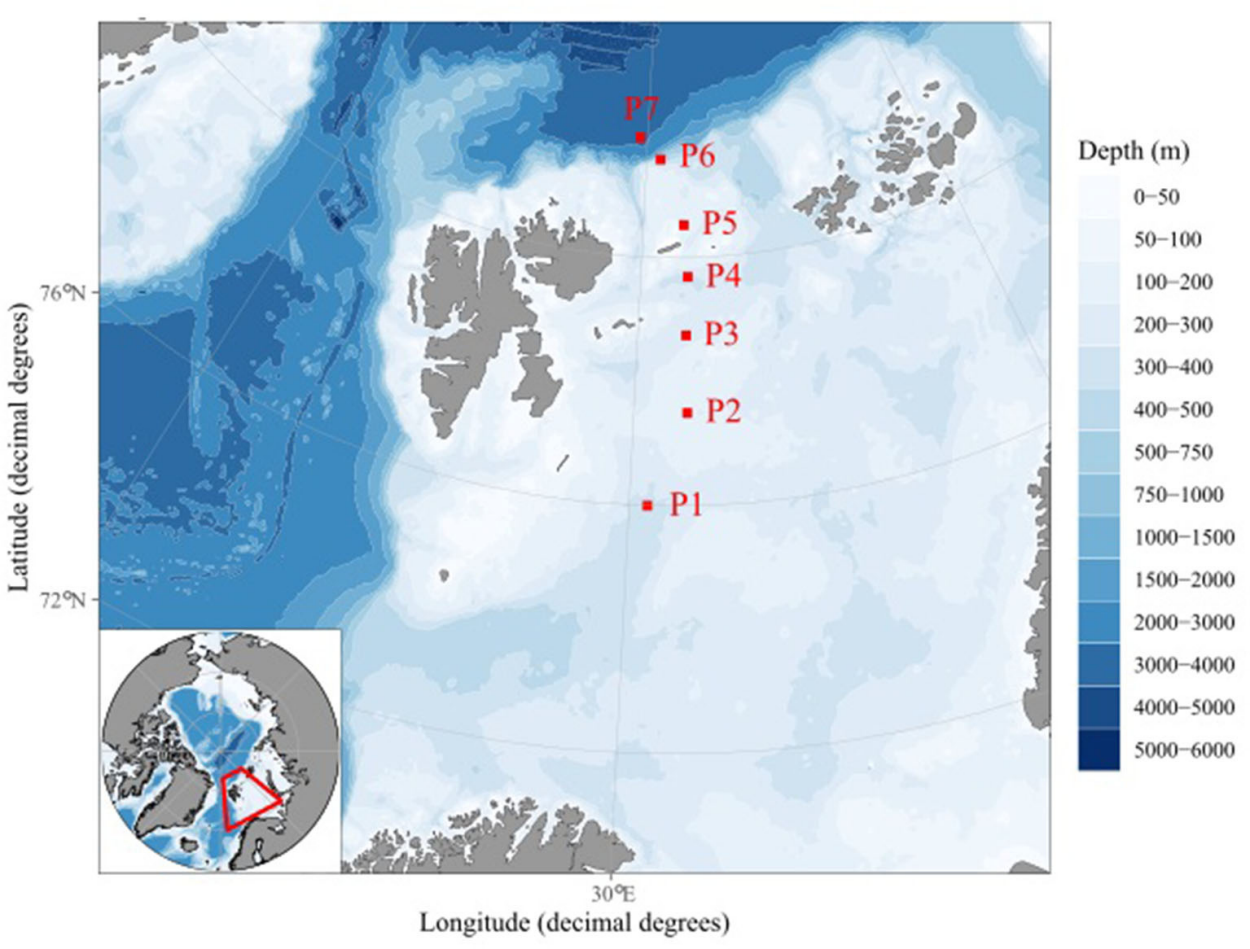
Map of the Nansen Legacy transect in the Barents Sea with stations P1 to P7 marked in red. The map was made using “ggOceanMaps” (Vihtakari, 2022).

Samples were collected during four seasonal sampling campaigns on the RV “Kronprins Haakon,” during different quarters (Q) of the year along the standard transect of the Nansen Legacy project in the northern Barents Sea east of Svalbard (stations P1–P7; Figure 1): Q3 (cruise 2019706) from 6 to 23 August 2019, Q4 (cruise 2019711) from 28 November to 17 December 2019, Q1 (cruise 2021703) from 2 to 24 March 2021, and Q2 (cruise 2021704) from 27 April to 20 May 2021, with Q1 representing “late winter,” Q2 “spring,” Q3 “summer,” and Q4 “early winter.” The stations were chosen to cover several factors of the Northern Barents Sea, such as Atlantic water influence (P1, P6, and P7), possible sea ice coverage (P5–P7), the Barents Sea shelf (P1–P5), the shelf break (P6), and the Arctic Basin (P7). All samples were taken as single samples using a Niskin rosette following the latest version of the Nansen Legacy protocol (The Nansen Legacy, 2021). Samples for the analyses of the bacterial and archaeal communities were collected at 10 m, the chlorophyll *a* max, or 20 m if no chlorophyll *a* max was found, 200 m, 1,000 m when possible, and 10 m above the sea floor. The differences in depth are due to slight differences in the sea floor depth around the station, which in combination with the CTD depth, drift, and other parameters during sampling leads to slight variation, especially at the deeper stations. Samples deeper than 50 m were defined as “sub-seasurface” based on the differences in the community composition compared to surface samples (0–50 m). All water samples were filtered using 0.22 μm Sterivex filters (Merck, Darmstadt, Germany), frozen immediately, and kept at −80°C until DNA extraction. During sampling campaigns, temperature, salinity, and the concentrations of phosphate, silicate, nitrate, and nitrite were measured (Chierici, 2021a,b; Jones et al., 2022a,b). In addition, the sea ice coverage and the days without ice cover were noted (Steer and Divine, 2023). Chl *a* concentrations were measured using a Turner Design Fluorometer from seawater after extraction from GFF filters using methanol. Bacterial and archaeal cell numbers were determined using a FACSCalibur (Becton Dickinson, Franklin Lake, US) flow cytometer according to Brussaard and co-workers (Marie et al., 1999; Brussaard, 2004). Aliquots of 100 μm were taken from 1.8 ml of glutaraldehyde-fixed seawater samples and diluted to concentrations expected to yield the most accurate flow cytometry counts. The dilutions were stained using SYBR Green I DNA dye and counted at a low flow rate of ~60 μl min-1 using a bi-parametric plot of green fluorescence (530/30) vs. side scatter (SSC,488/10) to differentiate cells. All samples are presented as numbers with standard errors.

### 2.2. DNA extraction and sequencing

DNA extractions were performed using the DNeasy Power Water Sterivex Kit (QIAGEN, Hilden, Germany) according to the manual. The extracted DNA was used for sequencing library construction, targeting the V4 region of the 16S rRNA genes of the bacterial and archaeal communities, using primers 515F−5′-GTGYCAGCMGCCGCGGTAA-3′ and 806R−5′-GGACTACNVGGGTWTCTAAT-3′ (Apprill et al., 2015; Parada et al., 2016). The libraries were sequenced using Illumina MiSeq technology with paired-end reads of 2 × 250 bp length at the Integrated Microbiome Resource in Halifax, Canada. The raw reads were deposited in the European Nucleotide Archive as project PRJEB57296.

### 2.3. Sequence analyses

The DADA2 pipeline was used in R to generate Amplicon sequence variants (ASVs) (Callahan et al., 2016). For this, primers were removed, the quality of the sequences was checked, and a static trim with 230 bp and 200 bp was conducted. The final ASVs were then generated after dereplication. Subsequently, the complementary reads were merged, chimeras were removed, and the taxonomy was assigned to the ASVs using a trained Silva database, based on the Silva release SSU Ref NR v138 (Quast et al., 2013). Thereafter, ASVs identified as mitochondria, chloroplasts, eukaryotes, < 4 sequences or < 1 × 10^−5^% of relative abundance, as well as samples with < 10.000 reads were removed (Supplementary material 1). The samples are presented with relative abundances with standard deviations if applicable.

All analyses were performed using R in RStudio (R Core Team, 2021) with the packages “tidyverse,” “phyloseq” for the analyses of the community data (McMurdie and Holmes, 2013; Wickham et al., 2019) and “ape” and “forcats” as supportive packages (Paradis and Schliep, 2019; Wickham, 2020). Visualizations were performed using the packages “ggplot2,” “scales,” and “patchwork” (Wickham, 2009; Lin Pedersen, 2020; Wickham and Seidel, 2020), while statistics were performed using the “vegan” and “mixOmics” packages (Rohart et al., 2017; Oksanen et al., 2020). An approximate maximum likelihood tree was calculated using FastTree2 (Price et al., 2010) based on alignments using the SINA aligner (Pruesse et al., 2012). A redundancy analysis (RDA) including an ANOVA test for the significance of different factors was conducted using “microbiomeSeq” (Ssekagiri et al., 2017). In addition, a partial least square regression (PLSR) analysis was performed using the “mixOmics” package (Rohart et al., 2017). Using PiCRUSt2 with standard parameters in bioconda, the metabolic potential of the different ASVs was inferred based on their location in a phylogenetic tree of fully sequenced organisms and the genomic assets of the closest relative in this tree (Grüning et al., 2018; Douglas et al., 2020). Using enzyme commission (EC)-numbers, the carbohydrate-active enzymes (CAZymes) belonging to the classes of auxiliary activities (AAs), glycoside hydrolases (GH), polysaccharide lyases (PL), carbohydrate esterases (CE), carbohydrate-binding modules (CBMs), and glycosyltransferase (GT) were extracted. CAZymes classified as GH and CBM were merged into GH. Similarly, marker genes, namely *nifH, nirS/nirK, norB, nosZ, narB, hzsA, amoA-pmoA, hao, nrfA, aprA, dsrA, cysH, soxB, mmoX, mcrA, psaA*, and *psbA* were used for the analyses (Supplementary materials 2, 3).

## 3. Results

### 3.1. Environmental variables

**Figure 2:**
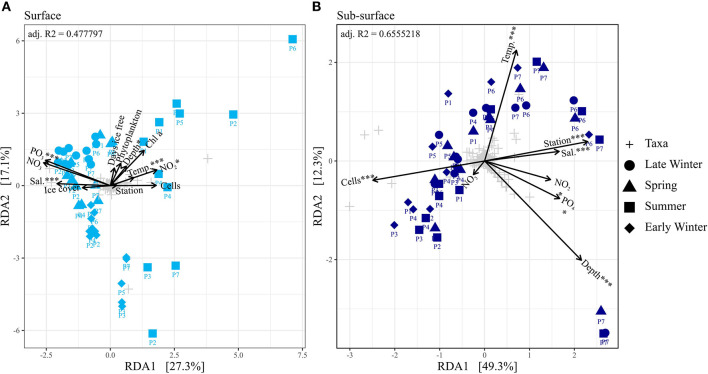
**(A, B)** Ordination plot of a redundancy analyses of the different seasons separated into surface and sub-seasurface samples. Environmental variables were abbreviated, and units were omitted for readability (Temp, Temperature; Sal, Salinity; Phytoplankton, Phytoplankton cell abundance; Cells, Bacterial and archaeal cell abundance). Chl a concentration, Ice cover and ice-free days were omitted in the RDA of the sub-seasurface samples. Significance code of the RDA is ^***^ = 0.001 and ^*^ < 0.05.

In early and late winter, as well as in spring, stations P2–P7 were ice covered, while in summer P4–P7 were covered. The ice cover ranged between 80 and 100% coverage. Station P1 was ice-free for 268 days in late winter, 324 days in spring, and 215 days in early winter. In summer, stations P1–P3 were ice-free for 89, 43, and 44 days, respectively. The salinity ranged ~34.5 ± 0.4 PSU, indicating the presence of polar water and warm polar water (Sundfjord et al., 2020). Lower salinities were measured on the surface of ice-covered stations, especially during summer and early winter, with the lowest value of 32.0 PSU recorded at 10 m depth at P5 during summer (Supplementary Figure 1 and Supplementary material 4). Thus, salinity is a significant (ANOVA; *p* = 0.001) factor explaining the bacterial and archaeal community composition on the surface, separating the summer from the rest of the seasons, as well as P6 and P7 from the other stations in the sub-seasurface (Figure 2). This separation is intensified by the differences in temperature (ANOVA; *p* = 0.001; Figure 2). Temperatures ranged from a maximum value of 4.72°C at 6 m at the southernmost station P1 during summer to −1.87°C at most depths at P3 during early winter (Supplementary Figure 1 and Supplementary material 4). Generally, temperatures were lower in surface waters than in the sub-seasurface, especially in ice-covered stations (Supplementary Figure 1).

Phosphate, silicate, and nitrate concentrations increased with depth (Supplementary Figure 1 and Supplementary material 4). Overall, phosphate concentrations ranged from 0.04 to 1.04 μmol L^−1^, silicate concentrations ranged from 0.20 to 12.7 μmol L^−1^, nitrate ranged from 0.00 to 15.3 μmol L^−1^, and nitrite ranged from 0.01 to 0.35 μmol L^−1^ (Supplementary Figure 1 and Supplementary material 4). Phosphate concentrations were highest in the deepest samples of stations P6 and P7. The highest nitrate values were found in spring, with elevated concentrations throughout the water column (Supplementary Figure 1 and Supplementary material 4). At the same time, nitrite concentrations were low at all stations, never exceeding 0.02 μmol L^−1^ (Supplementary Figure 1 and Supplementary material 4) but were still significant drivers for the community composition (*p* = 0.034 surface; *p* = 0.024 sub-seasurface; Figure 2). Chlorophyll *a* values were highest in the surface layers during spring, reaching 3.17 μg L^−1^ at 30 m at P6 and even 4.25 μg L^−1^ at 90 m at P7 (Supplementary Figure 1 and Supplementary material 4). The concentrations were low during summer, lower in early winter, and lowest concentrations were measured during late winter, when Chl *a* concentrations never exceeded 0.02 μg L^−1^ (Supplementary Figure 1 and Supplementary material 4).

### 3.2. Bacterial and archaeal cell numbers

The bacterial and archaeal cell numbers were generally higher in surface waters with an average of 2.9 × 10^5^ ± 5.1 × 10^4^ cells ml^−1^ than in the sub-seasurface with an average of 1.4 × 10^5^ ± 1.4 × 10^4^ cells ml^−1^ (Supplementary Figure 1). Lowest cell abundances were found in the deepest samples at stations P6 and P7, which significantly (*p* = 0.001) separated these samples from the rest of the sub-seasurface samples in the RDA (Figure 2; Supplementary Figure 1). They were overall highest during summer, especially in surface waters reaching 2.3 × 10^6^ cells ml^−1^ at 10 m depth at station P5 (Supplementary Figure 1). The cell abundance in surface waters was lower in early winter, at a minimum in late winter, and higher again in spring (Supplementary Figure 1). These changes were more prominent in the surface waters, with cell numbers being more stable in the sub-seasurface (Supplementary Figure 1 and Supplementary material 4).

### 3.3. Bacterial and archaeal community composition

**Figure 3:**
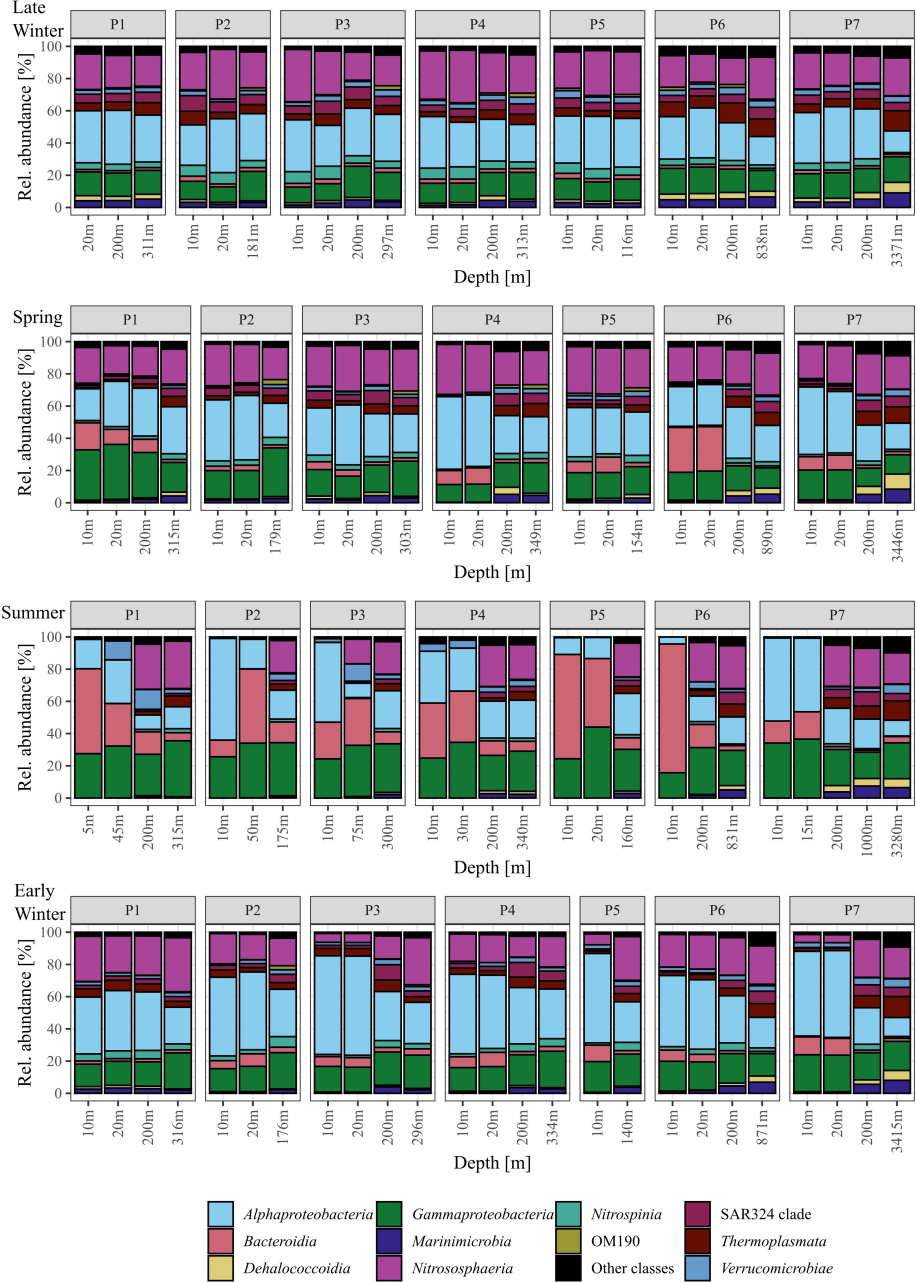
Relative abundance of the most abundant classes detected using 16S rRNA gene sequencing. Classes under 1% mean relative abundance are summed as “Other classes”.

Bacterial and archaeal communities from surface and sub-seasurface waters were markedly different in spring, summer, and early winter, but relatively similar in late winter. For the surface samples, the season was the most prominent factor differentiating between the samples, while for sub-seasurface samples, the location accounted for the most differences (Figure 2). The bacterial community was dominated by *Alphaproteobacteria* and *Gammaproteobacteria* with 55 ± 14 and 44 ± 7% combined *Proteobacteria* relative abundance in the surface and sub-seasurface (Figure 3). *Bacteroidia* were second with 14 ± 17 and 4 ± 5%. These were complemented by members of the SAR324 clade, *Verrucomicrobiota, Nitrospinota, Marinimicrobia, Planctomycetota*, and *Chloroflexi*, which all were more abundant in sub-seasurface waters than at the surface. These phyla accounted for ~74% of the community and were complemented by the archaeal phyla *Crenarchaea* and *Thermoplasmatota* which together accounted for ~24%. Both archaeal phyla were more abundant in sub-seasurface waters with 22 ± 4 and 6 ± 3%, than at the surface with 17 ± 11 and 3 ± 2%. In general, the sub-seasurface community was relatively stable throughout all four seasons, while the surface layer showed distinct changes in the bacterial and archaeal communities (Figure 3).

**Figure 4:**
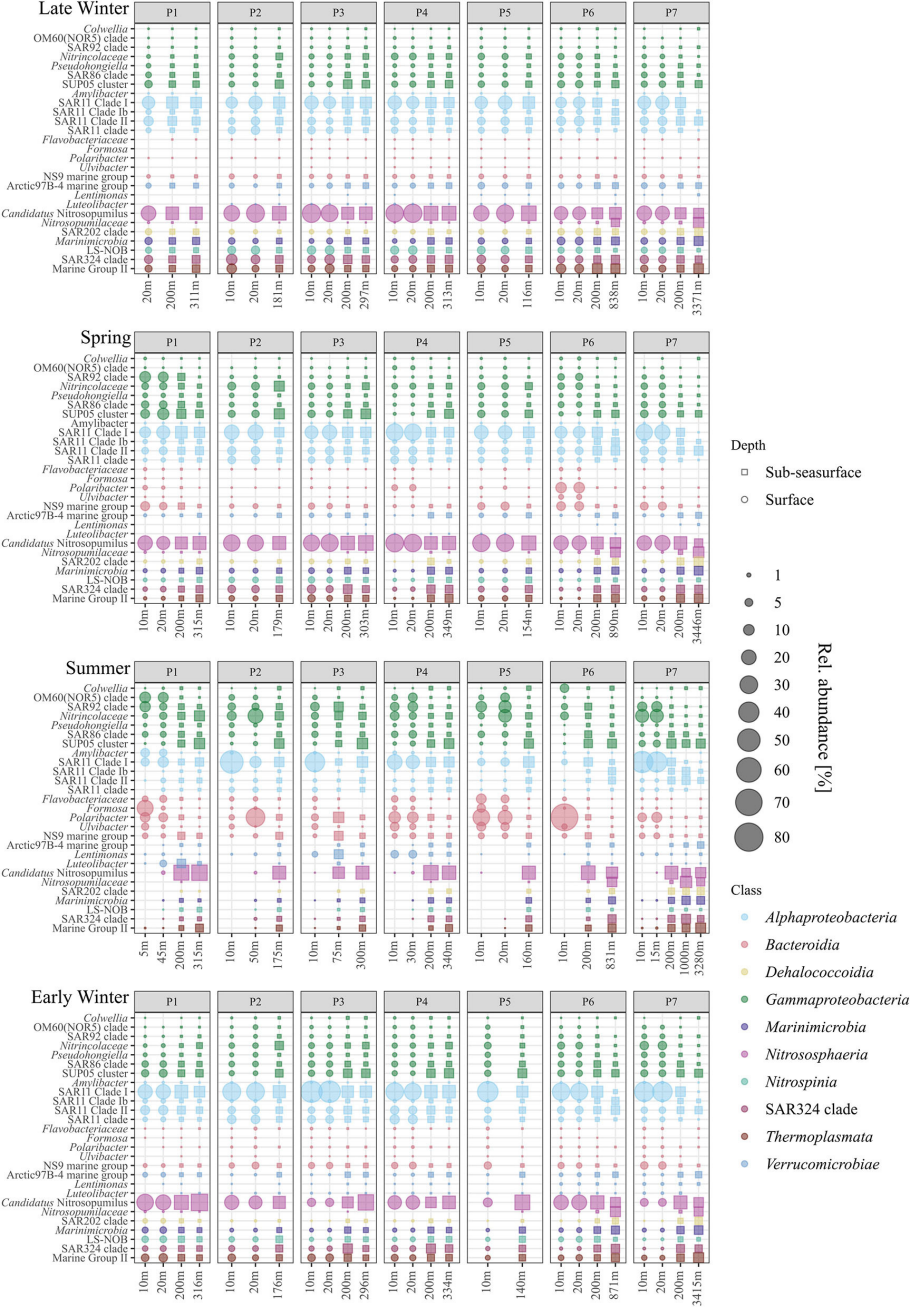
Relative abundance of the most abundant genera per class (color coded) for surface (•) and sub-seasurface (▪) waters of the stations P1 to P7 for 2021 (late winter, spring) and 2019 (summer, early winter).

At the surface, *Nitrosphaeria*, specifically *Cand*. Nitrosopumilus, accounted for the most prominent changes over the four seasons (Figure 3). While *Cand*. Nitrosopumilus was dominant during late winter and spring with 24 ± 6%, the relative abundance was < 0.2% during summer, before increasing again to 14 ± 8% during early winter, but with lower abundance at stations P3, P5, and P7 (~6%, Figure 4). This pattern is closely mirrored by *Nitrospinia*, which co-occurred with *Cand*. Nitrosopumilus (Figure 3). Within the *Nitrospinia*, members of the LS-NOB clade were dominant with 5 ± 1%, 1 ± 1%, >0.01%, and 2 ± 1% relative abundance during late winter, spring, summer, and early winter, respectively (Figure 4).

*Bacteroidia* showed opposite patterns to *Cand*. Nitrosopumilus by being low in late winter (2 ± 1%), increasing during spring (11 ± 8%), peaking at 37 ± 21% in summer, before decreasing again to 7 ± 3% in early winter (Figure 3). Among the *Bacteroidia*, members of the NS9 group were the most constant with average relative abundances between 1% in late winter and 4% in spring (Figure 4). All other dominant *Bacteroidia* were below 0.5% relative abundance during late winter, spring, and early winter, except for *Polaribacter* which were elevated at stations P1, P4, and P6 during spring (1%, 3%, and 10%; Figure 4). During summer, all these groups became more dominant. Especially *Polaribacter*, with an average abundance of 17% in surface waters, was the second most abundant genus in the dataset. At station P6, *Polaribacter* made up 72% of the community at this time (Figure 4). Similarly, *Formosa, Ulvibacter, Flavobacteriaceae*, and members of the NS9 clade were higher during summer but showed differences between different stations (Figure 4).

Similar dynamics with different taxa being abundant at different stations were found for *Gammaproteobacteria*. Overall, *Gammaproteobacteria* had the lowest abundance during late winter, were more abundant in spring, peaked in summer, and were less abundant again in early winter (13 ± 2%, 18 ± 7%, 30 ± 8%, and 17 ± 3%; Figure 3). Within the *Gammaproteobacteria*, members of the SUP05 clade were most abundant, although mostly in sub-seasurface waters, where the taxon accounted for more than 5% of the total community (Figure 4). In surface waters, *Nitrincolaceae, Pseudohongiella*, and members of the SAR86 and SAR92 clades were the most dominant gammaproteobacteria taxa (Figure 4). In this study, *Pseudohongiella* and members of the SAR86 clade were relatively stable throughout the seasons at 1 and 2 ± 1%, whereas *Nitrincolaceae* and members of the SAR92 and OM60(NOR5) clades varied more, all of them peaking during summer (9 ± 6%, 7 ± 3%, and 4 ± 4%; Figure 4). The standard deviation is the result of stations being highly variable during summer, when *Nitrincolaceae* were most abundant at P2, P5, and P7, members of the SAR92 clade at stations P1, P4, P5, and P7, and members of the OM60(NOR5) clade at P1, P4, and P5 (Figure 4). *Colwellia* was relatively abundant at 10 m depth at station P6 with 6% but was otherwise low (Figure 4).

*Alphaproteobacteria* was the most abundant class overall with 30 ± 3%, 34 ± 8%, and 30 ± 19% during late winter, spring, and summer, respectively, and higher during early winter with 49 ± 8% (Figure 3). The most dominant taxa within the *Alphaproteobacteria* belonged to the SAR11 group, being members of the SAR11 Clades I, Ib, II, and an unspecified SAR11 clade. While the latter three clades were stable with 2 ± 1%, 6 ± 3%, and 3 ± 1% during late winter, spring, and early winter, all were lower than 1% during summer (Figure 4). Members of the SAR11 Clade I were low during late winter and during spring with 15 ± 2% and 19 ± 5%, but very abundant, although with high variability between stations, during summer (e.g., 51%, 37%, and 45% at stations P2, P3, and P7). The highest relative abundance was found during early winter at 33 ± 9% (Figure 4). A single peak of *Amylibacter* (previously NAC11-7 clade) was found with 6% at P1 during summer (Figure 4).

Among the *Verrucomicrobiae*, the Arctic97B-4 marine group dominated, being stable ~3% in sub-seasurface waters, and 2% at the surface during late winter, but below the detection limit during summer (Figure 4). In this study, *Lentimonas* and *Luteolibacter* were more abundant in the sub-seasurface but also reached 5 and 4% in surface waters at stations P4 and P1 (Figure 4). These classes were complemented by members of the SAR202 and SAR324 clades, *Marinimicrobia*, and Marine Group II archaea of the *Thermoplasmata*, all of which were abundant in sub-seasurface bot lower than 0.1% in surface waters during summer (Figures 3, 4).

### 3.4. Inferred metabolic potential of the bacterial and archaeal community

**Figure 5:**
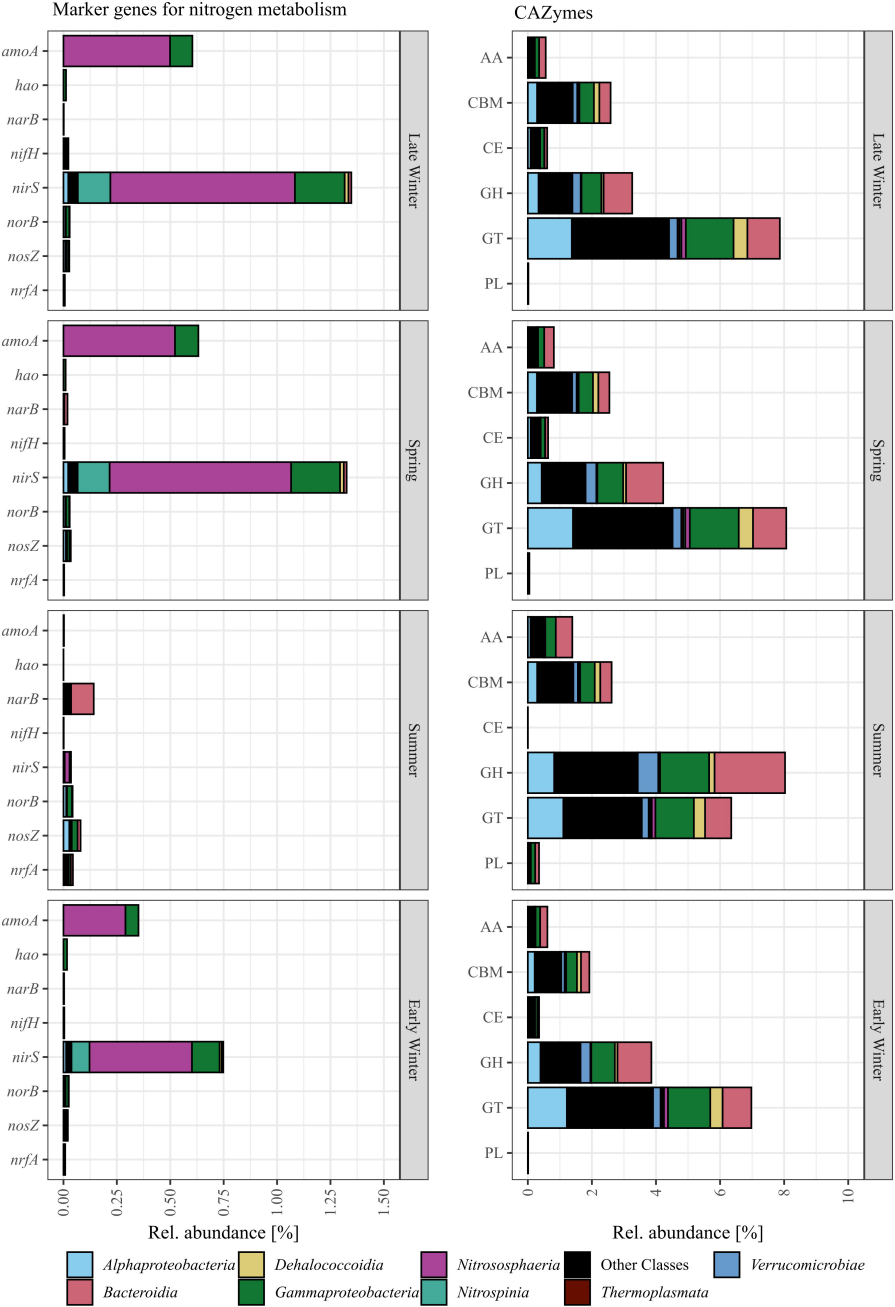
PiCRUSt2 inferred gene abundance of marker genes for nitrogen metabolisms (*amoA*, nitrification; *hao*, nitrification; *narB*, denitrification; *nifH*, nitrogen fixation; *nirS/nirK*, denitrification; *norB*, denitrification; *nosZ*, denitrification; *nrfA*, nitrate reduction to ammonium) and CAZymes summed by category (AA, auxiliary activities; CE, carbohydrate esterases; CBM, carbohydrate-binding modules; GH, glycoside hydrolases; GT, glycosyl transferase; PL, polysaccharide lyases) shown with their phylogenetic assignment. The gene abundance is shown as the sum of all surface samples per quarter of the year.

Using PiCRUSt2, CAZymes and marker genes for nitrogen-, sulfur-, and methane metabolism, as well as photosynthesis, were inferred from genomes of closely related strains deposited in the NCBI database. Photosynthesis marker genes, *soxB, napA, hzsA*, and *mmoX* were not detected, while the abundance of marker genes for methane and sulfur metabolisms was low (*mcrA* and dsrA*)* or showed constant abundance among the samples (*cysH* and *aprA*). Genes for nitrogen metabolisms and CAZymes showed similar abundance in sub-seasurface waters throughout the samples and are therefore not shown. Surface samples were summed by the gene for each season as a conservative approach to investigate the inferred genes. Genes for nitrogen fixation (*nifH*), nitrate reduction to ammonium (*nrfA*), and denitrification (*narB, norB*, and *nosZ*) were low, except for *narB* with 0.14% relative sequence abundance during summer, mostly affiliated to *Bacteroidia* (Figure 5). The most abundant genes were related to nitrification (*amoA-pmoA*) and denitrification (*nirS/nirK*), and were affiliated to *Nitrospinia, Nitrosphaeria*, mainly LS-NOB, and *Cand*. Nitrosopumilus and *Nitrosopumilaceae*, as well as *Gammaproteobacteria* (Figure 5). These genes were abundant during late winter and spring (0.6 and 1.4%, and 0.6 and 1.3%, for nitrification and denitrification, respectively; Figure 5). The abundance was < 0.1% during summer and higher again to 0.4% and 0.8% in early winter (Figure 5). Most *amoA-pmoA* genes are affiliated with taxa known to perform nitrification. Hence, we assume that the gene is mostly the ammonia monooxygenase coding *amoA* involved in nitrification. However, methanotrophic taxa, such as *Methyloprofundus*, were also found, implying that *pmoA* genes involved in methanotrophy are included in small amounts. Since these abundances are approximately two orders of magnitude smaller than the abundance of *amoA*, we here refer to both genes as *amoA*.

Within the groups of CAZymes, only glycosyl hydrolases and glycosyl transferases were high and showed distinct changes in abundance. Glycosyl transferases, mostly affiliated with *Alphaproteobacteria, Gammaproteobacteria, Bacteroidia*, and many less abundant classes, were high during all quarters of the year, but were higher during late winter and spring (~8%) as compared to 6.7% during summer and early winter (Figure 5). Glycosyl hydrolases, affiliated to *Alphaproteobacteria, Gammaproteobacteria, Bacteroidia, Verrucomicrobia*, and many less abundant classes, were lowest in early and late winter with 3.9 and 3.26%, but higher in spring and summer with 4.22, and 8.02% (Figure 5).

## 4. Discussion

During the Nansen Legacy project, early winter and summer were sampled in 2019, while late winter and spring were sampled in 2021. The communities representing early and late winter are relatively similar despite being sampled more than 1 year apart. In addition, the summers in 2019 and 2021 were relatively similar in environmental conditions as well as the bacterial and archaeal communities (Thiele et al., 2023). This is further supported by the occurrence and dominance of identical ASVs of *Cand*. Nitrosopumilus, *Polaribacter*, and the main ASV of SAR11 Clade 1a, the most dominant taxa, in all samples. Therefore, we consider this dataset as a useful representation of major seasonal variations in the bacterial and archaeal community of this marine Arctic area.

**Figure 6:**
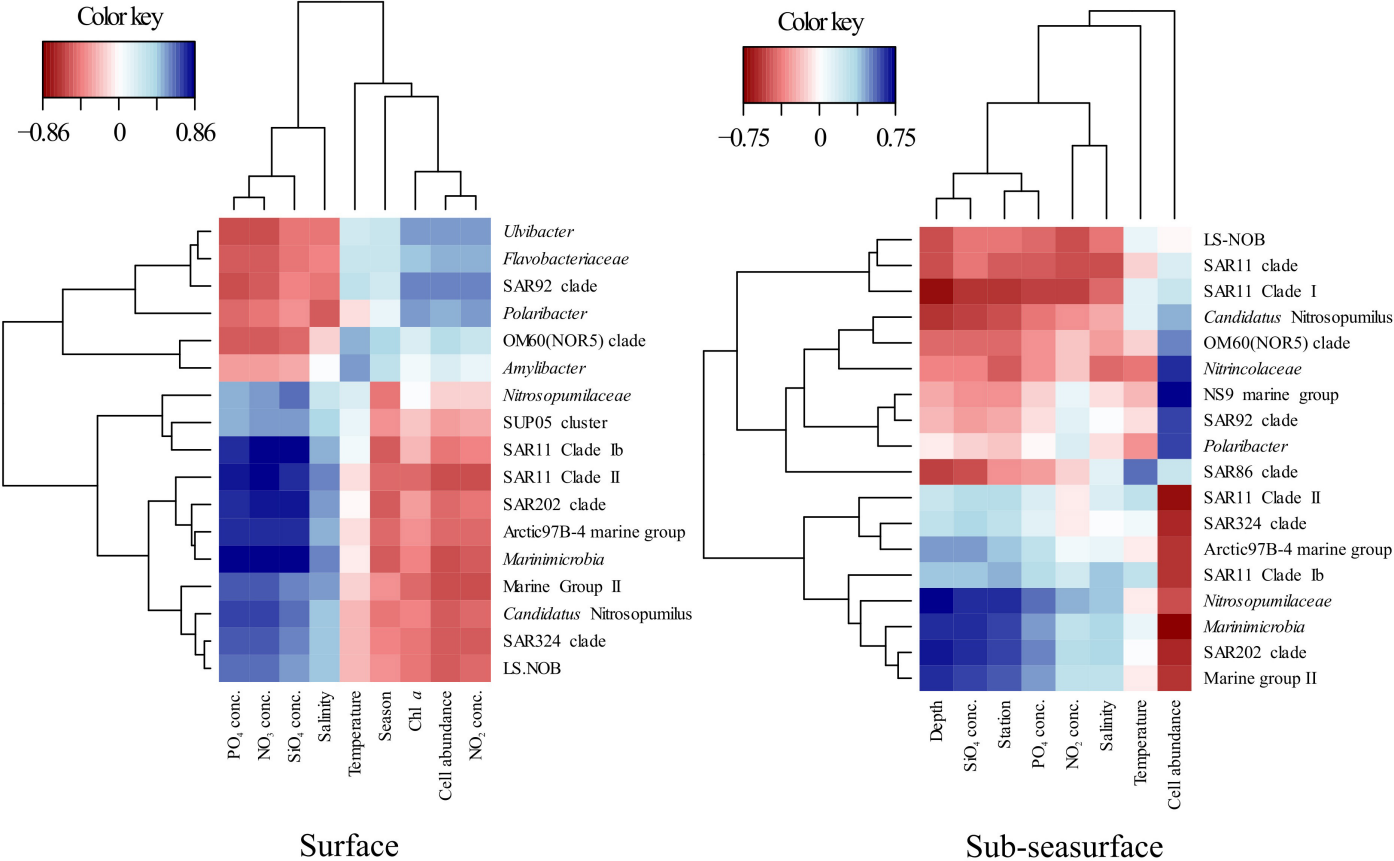
Partial least square regression (correlation coefficient >0.5) between environmental parameters and the relative abundance of the dominant bacterial and archaeal groups in the surface and sub-sea surface waters showing the environmental drivers of the community structure.

Except during late winter, the bacterial and archaeal communities in surface and sub-seasurface samples were different. In the sub-seasurface, the main differentiating factors between samples were the location of the station, salinity, depth, temperature, phosphate and nitrite concentrations, and cell abundance, with P6 and P7 being the most different from other stations. These stations are deeper and have different water bodies in the deep waters, which explains the differences in temperature and salinity, as well as the higher phosphate and nitrite values. Generally, the abundance of bacterial and archaeal cells decreases with depth in the ocean thus explaining the lower cell numbers at the deep stations. The main environmental variables correlating with the sub-seasurface main bacterial and archaeal taxa, besides stations and depth, were higher phosphate and nitrite concentrations, lower cell abundance, temperatures, and higher salinity (Figure 6). However, the communities showed much less variation between the different seasons than in the surface waters. The community in the sub-seasurface samples was dominated by *Cand*. Nitrosopumilus, members of the SAR11 clades, *Marinimicrobia*, members of the SAR324 clade, and the LS-NOB clade within the *Thermoplasmata*. Similar communities have been found at different times in areas around Svalbard thus implying that these taxa broadly resemble the bacterial and archaeal community of sub-seasurface Arctic waters (Wilson et al., 2017; Müller et al., 2018; de Sousa et al., 2019).

In surface waters, cell numbers were low from late winter to spring, were highest in summer, and decreased again toward early winter, indicating a seasonality of the bacterial and archaeal community. This was confirmed by partial least square regression analyses determining the season, the correlated Chl *a* and nitrite concentrations, and cell abundance, as the main driving factors of the surface community composition (Figure 6). The surface water undergoes seasonal changes with constant daylight and higher temperatures in summer leading to ice melt and consequently slightly lower surface water salinity. At stations P1, P6, and P7, these changes could be an indicator of the influx of Atlantic waters into the Arctic by the West Spitsbergen Current at these stations (Loeng, 1991; Lind and Ingvaldsen, 2012; Asbjørnsen et al., 2020). However, following the “everything is everywhere” -hypothesis (O'Malley, 2008), large changes in the bacterial and archaeal community due to waters with only slightly different environmental conditions are unlikely. Secondary effects, such as the transport of phytoplankton blooms with the incoming waters, however, could be a factor for changes in the community composition (Oziel et al., 2020).

### 4.1. Spring

The environmental conditions during April/May 2021 were characterized by the highest concentrations of nitrate and Chl *a* in the waters. While the high nitrate concentration is a result of vertical mixing and, potentially, nitrification in surface waters during winter, the onset of the phytoplankton spring bloom in April and May provides carbon and energy to the whole Arctic marine ecosystem (Degerlund and Eilertsen, 2010; Assmy et al., 2017), including bacterial and archaeal communities (Teeling et al., 2012, 2016). This leads to a succession, mostly driven by bacterial groups specialized for carbon utilization, such as *Gammaproteobacteria* and *Bacteroidia*, hence explaining the higher abundance of these groups in the spring. Members of the SAR92 clade, *Polaribacter*, and members of the NS9 clade had high abundances during spring, marking the onset of the succession. Members of the SAR92 clade were found as early responders to an Antarctic phytoplankton bloom (Liu et al., 2020), and *Polaribacter* has globally been found in co-occurrence with phytoplankton blooms (Gomez-Pereira et al., 2010; Thiele et al., 2012, 2015; Xing et al., 2015; Teeling et al., 2016). The NS9 clade has, however, not been found to correlate with phytoplankton blooms in polar environments.

### 4.2. Summer

The summer situation is reflected by the communities found during a late phytoplankton bloom situation (Thiele et al., 2023). In this study, *Nitrincolaceae* (formerly *Oceanospirillaceae*), members of the SAR92 and OM60(NOR5) clades were most abundant among the *Gammaproteobacteria*, while *Polaribacter, Formosa, Ulvibacter*, and *Flavobacteriaceae* ASVs were abundant members of the *Bacteroidia*. The abundance of these two classes indicates waters with elevated concentrations of phytoplankton-derived carbon sources (Simon et al., 1999; Puddu et al., 2003; Gomez-Pereira et al., 2010; Teeling et al., 2012, 2016; Thiele et al., 2012). *Nitrincolaceae* are known for high diversity in carbon utilization metabolisms and to rapidly respond to increased carbon availability. The group has been found in phytoplankton blooms in Antarctica, the Arctic, and Arctic sea ice (Mori et al., 2019; Liu et al., 2020; Mönnich et al., 2020; Park et al., 2020; Wietz et al., 2021; Thiele et al., 2022). SAR92 clade members remained active in blooms, and members of the OM60(NOR5) clade have been found to correlate with phytoplankton blooms (Yang et al., 2015). In addition, a similar community of *Bacteroidia* consisting of *Polaribacter, Formosa*, NS9 clade, and further unidentified *Flavobacteriaceae* ASVs have been found in a spring–summer bloom in the Fram Strait west of Svalbard (Wietz et al., 2021). Generally, *Bacteroidia* co-occur in abundance with phytoplankton blooms, exploiting the derived carbon sources due to their multitude of carbon degradation pathways and carbohydrate-active enzyme content (Gomez-Pereira et al., 2010; Reintjes et al., 2019, 2020). Interestingly, a single peak of the *Amylibacter*, a taxon also found during summer to fall transitions west of Svalbard (Wietz et al., 2021), was found at station P1 indicating that this station might be in a later stage of the succession than the other stations, which is supported by lower Chl *a* values. Generally, the stations showed higher variability during summer, pointing toward different bloom stages, potentially due to ice coverage-mediated variability in the onset and strength of the spring phytoplankton bloom (Hunt et al., 2013; Silva et al., 2021). As the bloom progresses, different carbon sources are available, thus defining the nutritional habitat of the station and consequently determining the diversity and community structure of the bacterial and archaeal community (Teeling et al., 2012, 2016). This is supported by the higher abundance of GH genes during summer, indicating that species with more CAZyme coding genes were abundant during summer. CAZymes, and specifically glycoside hydrolases are enzymes involved in the degradation of carbohydrates. GHs are a widespread group targeting glycosidic bonds of substrates ranging from monosaccharides to polysaccharides and complex sugar components, e.g., glucans, fucans, mannans, or cellulose (Teeling et al., 2016). By that, the occurrence of different GHs can give hints toward the carbohydrate preference of the different taxa, thus explaining the succession within the bacterial and archaeal community. However, since the inferences made by PiCRUSt2 should only be used as indications, we have to refrain from detailed analyses of the different CAZyme groups.

Interestingly, *Cand*. Nitrosopumilus were substantially lower in surface waters during summer, although being abundant in the sub-seasurface throughout the year, and in surface samples during winter and spring. The same pattern has been found previously in Arctic water and ice and is attributed to the photoinhibition of ammonia oxidation (Collins et al., 2010; Grzymski et al., 2012; Wilson et al., 2017; Müller et al., 2018; de Sousa et al., 2019; Thiele et al., 2022).

### 4.3. Early and late winter

The transition from summer to winter, including fall was not fully covered by our sampling, therefore it remains speculative if a second phytoplankton bloom occurred and provided additional carbon sources to the microbial loop. Such fall blooms have previously been reported for the southern Barents Sea (Ardyna et al., 2014; Silva et al., 2021). In early winter 2019, the bacterial and archaeal community had transitioned back to a “winter state” with a low abundance of *Bacteroidia* and *Gammaproteobacteria* and a high abundance of *Alphaproteobacteria*. The decrease of *Bacteroidia* and *Gammaproteobacteria* implies that the succession based on phytoplankton-derived carbon was over and that the remaining carbon sources in the surface waters did not provide favorable conditions for these classes. Thus, *Nitrincolaceae*, together with members of the SAR86 and SUP05 clades, were the most dominant *Gammaproteobacteria*, all of which have been found in rather oligotrophic/winter communities in polar environments (Thiele et al., 2012, 2022; Wietz et al., 2021). Members of the SAR11 clade became dominant, possibly due to a decrease of other groups, as SAR11 clade members dominate the “base community” in marine and specifically polar waters (Morris et al., 2002; Schattenhofer et al., 2009; Thiele et al., 2012; Wilson et al., 2017; Wietz et al., 2021). In addition, *Cand*. Nitrosopumilus showed high abundance in the surface waters of some stations.

In late winter, *Cand*. Nitrosopumilus and members of the SAR11 clade were the most abundant taxa, and the surface layer closely resembled the sub-seasurface waters, with *Marinimicrobia*, members of the SAR202 and SAR324 clades, members of the Arctic97B-4 marine group, and the LS-NOB clade present at all depths, thus representing the winter community (Wilson et al., 2017; Müller et al., 2018). The co-occurrence of *Cand*. Nitrosopumilus, oxidizing ammonia to nitrite, and members of the LS-NOB clade (*Nitrospinia*) potentially completing the nitrification process, hints toward a chemoautotrophic community (Könneke et al., 2005; Luecker et al., 2013; Reji et al., 2019; Wietz et al., 2021; Rasmussen and Francis, 2022). This community oxidizes ammonia residuals derived from the decay of the spring/summer phytoplankton bloom, thus contributing to the replenishment of the nitrate pool toward spring. The high abundance of *amoA* genes during late winter and spring, which are affiliated with *Cand*. Nitrosopumilus, *Nitrosopumilaceae*, and members of the LS-NOB clade, supports this hypothesis. However, the abundance of *nirS/nirK* genes affiliated to *Cand*. Nitrosopumilus, *Nitrospinia*, and *Gammaproteobacteria* indicate a truncated form of denitrification, as full denitrification would require anoxic conditions, and the respective genes were not found. Hence, only the first two steps of denitrification might be conducted (Graf et al., 2014).

In conclusion, we are here presenting the bacterial and archaeal community of spring, summer, and early and late winter, sampled over a transect covering the northern Barents Sea east of Svalbard and into the Arctic Ocean. A shift in the community occurred from a winter state dominated by members of the SAR11 clade and a community of nitrifiers, namely *Cand*. Nitrosopumilus and LS-NOB (*Nitrospinia*), to spring and summer, with a succession of carbon-utilizing *Gammaproteobacteria* and *Bacteroidia*, back to an early winter state, with the re-establishment of ammonium oxidizers, and potential prevalence of chemoautotrophic metabolisms. The overarching Pattern observed in our year-round sampling from the northern barents sea resembles findings from an automated sampler in the Fram Straight west of Svalbard, where a similar shift of metabolisms was observed, although partly by different taxa (Wietz et al., 2021). Our study corroborates that spring and summer carbon cycling and winter nitrogen cycling are important processes driving the microbial ecology of Arctic marine ecosystems, and although annual variations are to be expected, it provides a baseline for future study on these processes in response to the effects of climate change. Specifically, sea ice coverage and the correlated variability of the phytoplankton bloom timing and strength might become a factor for the bacterial and archaeal community and thereby carbon cycling in the Northern Barents Sea.

## Data availability statement

The datasets presented in this study can be found in online repositories. The names of the repository/repositories and accession number(s) can be found in the article/Supplementary material.

## Author contributions

SThi analyzed the sequences and wrote the manuscript. STho, KS, EP, and OM conducted the laboratory preparations. AV, GB, and OM commented extensively on the manuscript. All authors contributed in the writing process.

## Supplementary material

Supplementary material 1 Table of sequence counts and relative abundance [%] of ASVs after the removal of ASVs with total abundances < 4. The samples are named with the time of the year (late winter, early winter, spring, and summer), station (P1–P7), and depth in meters, e.g., late winter_P1_0020 m.

Supplementary material 2 List of CAZymes used in this study.

Supplementary material 3 List of marker genes by metabolism used in this study.

Supplementary material 4 Table of environmental parameters measured at the different stations and depth.

Supplementary Figure S1 Depth profiles of the environmental variables measured during late winter (□), spring (Δ), summer (°), and early winter (∇). Column one depicts the temperature (blue) and the salinity (red), column two depicts the concentrations of phosphate (turquoise), silicate (orange), nitrate (dark yellow), and nitrite (yellow), column three depicts the chlorophyll *a* concentration (green), and column four depicts the abundance of bacterial and archaeal cells (black). Missing values were interpolated when possible, using a simple mean of the value above and below in the depth profile.
