# Supplementary material for: Seasonality of the bacterial and archaeal community composition of the Northern Barents Sea
Source: Front Microbiol. 2023 Jul 7;14:1213718. doi: 10.3389/fmicb.2023.1213718 (PMC10360405; doi:10.3389/fmicb.2023.1213718)
Supplement: Supplementary material 3 — List of marker genes by metabolism used in this study. [file Data_Sheet_1.PDF]

Carbohydrate-binding module (CBM)

EC-number	Name
EC:3.1.3.16	Protein-serine/threonine phosphatase
EC:2.4.1.21	Starch synthase
EC:2.4.1.18	1,4-α-glucan branching enzyme
EC:2.4.1.25	4-α-glucanotransferase
EC:3.1.1.73	Feruloyl esterase

Carbohydrate esterase (CE)

EC-number	Name
EC:3.5.1.108	UDP-3-O-acyl-N-acetylglucosamine deacetylase
EC:3.2.1.4	Cellulase
EC:3.5.1.25	N-acetylglucosamine-6-phosphate deacetylase
EC:3.2.1.55	Non-reducing end α-L-arabinofuranosidase
EC:3.1.1.3	Triacylglycerol lipase
EC:3.2.1.40	α-L-rhamnosidase
EC:3.1.1.74	Cutinase
EC:3.2.1.8	Endo-1,4-β-xylanase
EC:3.5.1.115	Mycothiol S-conjugate amidase
EC:3.5.1.105	Chitin disaccharide deacetylase
EC:2.3.1.20	Diacylglycerol O-acyltransferase
EC:3.1.1.11	Pectinesterase
EC:3.1.1.73	Feruloyl esterase
EC:4.2.2.2	Pectate lyase
EC:3.1.1.41	Cephalosporin-C deacetylase
EC:2.3.1.122	Trehalose O-mycolyltransferase

Glycosyl hydrolase (GH)

EC-number	Name
EC:3.2.1.1	α-amylase
EC:3.2.1.10	Oligo-1,6-glucosidase
EC:3.2.1.122	Maltose-6-phosphate glucosidase
EC:3.2.1.123	Endoglycoceramidase
EC:3.2.1.135	Neopullulanase
EC:3.2.1.139	α-glucuronidase
EC:3.2.1.14	Chitinase
EC:3.2.1.141	4-α-D-{(1→4)-α-D-glucano}trehalose trehalohydrolas
EC:3.2.1.166	Heparanase
EC:3.2.1.17	Lysozyme
EC:3.2.1.170	Mannosylglycerate hydrolase
EC:3.2.1.172	Unsaturated rhamnogalacturonyl hydrolase
EC:3.2.1.177	α-D-xyloside xylohydrolase

EC:3.2.1.18	Exo-α-sialidase
EC:3.2.1.180	Unsaturated chondroitin disaccharide hydrolase
EC:3.2.1.183	UDP-*N*-acetylglucosamine 2-epimerase (hydrolysing)
EC:3.2.1.184	UDP-*N*,*N′*-diacetylbacillosamine 2-epimerase (hydrolysing)
EC:3.2.1.20	α-glucosidase
EC:3.2.1.21	β-glucosidase
EC:3.2.1.22	α-galactosidase
EC:3.2.1.23	β-galactosidase
EC:3.2.1.24	α-mannosidase
EC:3.2.1.25	β-mannosidase
EC:3.2.1.26	β-fructofuranosidase
EC:3.2.1.28	α,α-trehalase
EC:3.2.1.3	Glucan 1,4-α-glucosidase
EC:3.2.1.31	β-glucuronidase
EC:3.2.1.37	β-xylosidase
EC:3.2.1.4	Endo-β-1,4-glucanase / cellulase
EC:3.2.1.40	α-L-rhamnosidase
EC:3.2.1.41	Pullulanase
EC:3.2.1.45	β-glucosylceramidase
EC:3.2.1.51	α-L-fucosidase
EC:3.2.1.52	β-N-acetylhexosaminidase
EC:3.2.1.55	α-L-arabinofuranosidase
EC:3.2.1.58	Glucan β-1,3-glucosidase
EC:3.2.1.6	Endo-1,3(4)-β-glucanase
EC:3.2.1.65	Levanase
EC:3.2.1.70	Glucan 1,6-α-glucosidase
EC:3.2.1.78	Mannan endo-β-1,4-mannosidase
EC:3.2.1.8	Endo-β-1,4-xylanase
EC:3.2.1.80	Fructan β-fructosidase
EC:3.2.1.81	β-agarase
EC:3.2.1.85	6-phospho-β-galactosidase
EC:3.2.1.86	6-phospho-β-glucosidase
EC:3.2.1.89	Arabinogalactan endo-β-1,4-galactanase
EC:3.2.1.91	Cellulose β-1,4-cellobiosidase
EC:3.2.1.93	α,α-phosphotrehalase
EC:3.2.1.96	Endo-β-N-acetylglucosaminidase
EC:3.2.1.99	Arabinan endo-1,5-α-L-arabinanase

Glycosyltransferase (GT)

EC-number	Name
EC:2.4.1.1	Glycogen phosphorylase
EC:2.4.1.11	Glycogen(starch) synthase
EC:2.4.1.12	Cellulose synthase (UDP-forming)
EC:2.4.1.129	Peptidoglycan glycosyltransferase

EC:2.4.1.13	Sucrose synthase
EC:2.4.1.14	Sucrose-phosphate synthase
EC:2.4.1.144	β-14-mannosyl-glycoprotein 4-β-N-acetylglucosaminyltransferase
EC:2.4.1.15	α, α-trehalose-phosphate synthase (UDP-forming)
EC:2.4.1.182	Lipid-A-disaccharide synthase
EC:2.4.1.208	Diglucosyl diacylglycerol synthase (1,2-linking)
EC:2.4.1.21	Starch synthase
EC:2.4.1.212	Hyaluronan synthase
EC:2.4.1.213	Glucosylglycerol-phosphate synthase
EC:2.4.1.227	Undecaprenyldiphospho-muramoylpentapeptide β-N-acetylglucosaminyltransferase
EC:2.4.1.245	α, α-trehalose synthase
EC:2.4.1.252	GDP-mannose:cellobiosyl-diphosphopolyprenol α-mannosyltransferase
EC:2.4.1.266	Glucosyl-3-phosphoglycerate synthase
EC:2.4.1.276	Zeaxanthin glucosyltransferase
EC:2.4.1.287	Rhamnopyranosyl-N-acetylglucosaminyl-diphospho-decaprenol β-1,4/1,5-galactofuranosyltransferase
EC:2.4.1.288	Galactofuranosylgalactofuranosylrhamnosyl-N-acetylglucosaminyl-diphospho-decaprenol β-1,5/1,6-galactofuranosyltransferase
EC:2.4.1.289	N-acetylglucosaminyl-diphospho-decaprenol L-rhamnosyltransferase
EC:2.4.1.57	Phosphatidylinositol α-mannosyltransferase
EC:2.4.1.58	Lipopolysaccharide glucosyltransferase I
EC:2.4.1.80	Ceramide glucosyltransferase
EC:2.4.1.83	Dolichyl-phosphate β-D-mannosyltransferase
EC:2.4.2.43	Lipid IV(A) 4-amino-4-deoxy-L-arabinosyltransferase
EC:2.4.99.18	Dolichyl-diphosphooligosaccharide--protein glycotransferase
EC:3.1.3.12	Trehalose-phosphatase

Polysaccharide lyase (PL)

EC-number	Name
EC:4.2.2.3	Mannuronate-specific alginate lyase
EC:4.2.2.2	Pectate lyase
EC:4.2.2.5	Chondroitin AC lyase
EC:4.2.2.1	Hyaluronate lyase
EC:4.2.2.20	Chondroitin-sulfate-ABC endolyase
EC:4.2.2.21	Chondroitin-sulfate-ABC exolyase
EC:4.2.2.23	Rhamnogalacturonan endolyase
EC:4.2.2.6	Oligogalacturonide lyase
