# Supplementary material for: Seasonality of the bacterial and archaeal community composition of the Northern Barents Sea
Source: Front Microbiol. 2023 Jul 7;14:1213718. doi: 10.3389/fmicb.2023.1213718 (PMC10360405; doi:10.3389/fmicb.2023.1213718)
Supplement: Supplementary material 4 — Table of environmental parameters measured at the different stations and depth. [file Data_Sheet_2.PDF]

Nitrogen metabolism

EC-number	Gene	Name	Pathway
EC:1.18.6.1	*nifH*	Nitrogenase reductase	Nitrogen fixation
EC:1.7.2.1	*nirS/nirK*	Nitrite reductase	Denitrification
EC:1.7.2.5	*norB*	Copper nitrite reductase	Denitrification
EC:1.7.2.4	*nosZ*	Nitric oxyde reductase	Denitrification
EC:1.7.7.2	*narB*	Nitrous oxide reductase	Assimilatory nitrate reduction
EC:1.7.2.7	*hzsA*	Periplasmatic nitrate reductase	Annamox
EC:1.7.2.6	*hao*	Hydrazine synthase	Nitrification
EC:1.14.99.39	*amoA*	Ammonia monooxygenase	Nitrification
EC:1.7.2.2	*nrfA*	Nitrite reductase	Dissimilatory nitrate reduction to ammonium

Sulfur metabolism

EC-number	Gene	Name	Pathway
EC:1.8.99.2	*aprA*	Adenylyl-sulfate reductase	Dissimilatory sulphate reduction
EC:1.8.99.5	*dsrA*	Dissimilatory sulfate reductase	Dissimilatory sulphite reduction
EC:1.8.4.8	*cysH*	Phosphoadenylyl-sulfate reductase	Assimilatory sulphate reduction
EC:3.1.6.20	*soxB*	Sulphate thiohydrolase	Sulphur oxydation

Methane metabolism

EC-number	Gene	Name	Pathway
EC:1.14.18.3	*pmoA*	Particulate methane monooxygenase	Aerobic methane oxydation
EC:1.14.13.25	*mmoX*	Methane monooxygenase	Aerobic methane oxydation
EC:3.1.21.-	*mcrA*	Coenzyme M methyl reductase	Methanogenesis

Photosynthesis

EC-number	Gene	Name	Pathway
EC:1.97.1.12	*psaA*	Reaction center PS I	Photosynthesis
EC:1.10.3.9	*psbA*	Reaction center PS II D1	Photosynthesis
