# Supplementary figures and images for: Seasonality of the bacterial and archaeal community composition of the Northern Barents Sea
Source: Front Microbiol. 2023 Jul 7;14:1213718. doi: 10.3389/fmicb.2023.1213718 (PMC10360405; doi:10.3389/fmicb.2023.1213718)

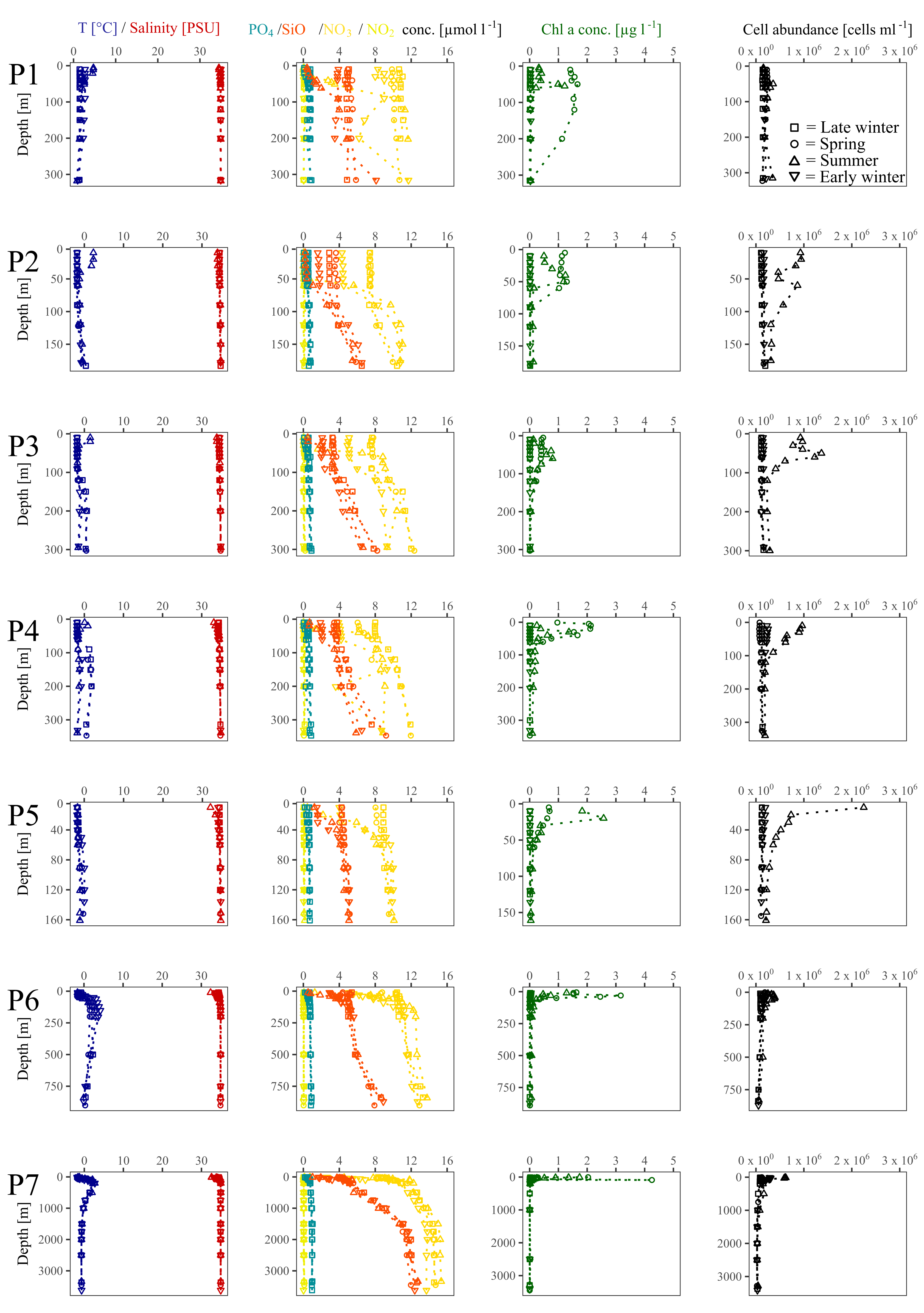

Supplement: Supplementary Figure S1 — Depth profiles of the environmental variables measured during late winter (□), spring (Δ), summer (°), and early winter (∇). Column one depicts the temperature (blue) and the salinity (red), column two depicts the concentrations of phosphate (turquoise), silicate (orange), nitrate (dark yellow), and nitrite (yellow), column three depicts the chlorophyll a concentration (green), and column four depicts the abundance of bacterial and archaeal cells (black). Missing values were interpolated when possible, using a simple mean of the value above and below in the depth profile. [file Image_1.PNG]
